# Compliant Substrates Enhance Macrophage Cytokine Release and NLRP3 Inflammasome Formation During Their Pro-Inflammatory Response

**DOI:** 10.3389/fcell.2021.639815

**Published:** 2021-03-29

**Authors:** Joan-Carles Escolano, Anna V. Taubenberger, Shada Abuhattum, Christine Schweitzer, Aleeza Farrukh, Aránzazu del Campo, Clare E. Bryant, Jochen Guck

**Affiliations:** ^1^Biotechnology Center, Center for Molecular and Cellular Bioengineering, Technische Universität Dresden, Dresden, Germany; ^2^Max Planck Institute for the Science of Light & Max-Planck-Zentrum für Physik und Medizin, Erlangen, Germany; ^3^INM – Leibniz-Institut für Neue Materialien, Saarbrücken, Germany; ^4^Department of Veterinary Medicine, University of Cambridge, Cambridge, United Kingdom

**Keywords:** innate immunity, macrophages, mechanosensing, substrate stiffness, NLRP3 inflammasome, ASC, actomyosin contractility

## Abstract

Immune cells process a myriad of biochemical signals but their function and behavior are also determined by mechanical cues. Macrophages are no exception to this. Being present in all types of tissues, macrophages are exposed to environments of varying stiffness, which can be further altered under pathological conditions. While it is becoming increasingly clear that macrophages are mechanosensitive, it remains poorly understood how mechanical cues modulate their inflammatory response. Here we report that substrate stiffness influences the expression of pro-inflammatory genes and the formation of the NLRP3 inflammasome, leading to changes in the secreted protein levels of the cytokines IL-1β and IL-6. Using polyacrylamide hydrogels of tunable elastic moduli between 0.2 and 33.1 kPa, we found that bone marrow-derived macrophages adopted a less spread and rounder morphology on compliant compared to stiff substrates. Upon LPS priming, the expression levels of the gene encoding for TNF-α were higher on more compliant hydrogels. When additionally stimulating macrophages with the ionophore nigericin, we observed an enhanced formation of the NLRP3 inflammasome, increased levels of cell death, and higher secreted protein levels of IL-1β and IL-6 on compliant substrates. The upregulation of inflammasome formation on compliant substrates was not primarily attributed to the decreased cell spreading, since spatially confining cells on micropatterns led to a reduction of inflammasome-positive cells compared to well-spread cells. Finally, interfering with actomyosin contractility diminished the differences in inflammasome formation between compliant and stiff substrates. In summary, we show that substrate stiffness modulates the pro-inflammatory response of macrophages, that the NLRP3 inflammasome is one of the components affected by macrophage mechanosensing, and a role for actomyosin contractility in this mechanosensory response. Thus, our results contribute to a better understanding of how microenvironment stiffness affects macrophage behavior, which might be relevant in diseases where tissue stiffness is altered and might potentially provide a basis for new strategies to modulate inflammatory responses.

## Introduction

Macrophages are innate immune cells responsible for engulfing microbes and cell debris and orchestrating inflammatory responses to maintain tissue homeostasis. While patrolling within different organs and tissues, macrophages are exposed not only to multiple biochemical signals but also to mechanical cues, including tissue stiffness. For instance, microglia residing in the human brain are exposed to shear moduli of a few hundred Pa (Sack et al., 2008; Christ et al., 2010; Elkin et al., 2010), alveolar macrophages in the lung to a Young’s modulus of 2 kPa (Booth et al., 2012), macrophages within dermal tissue to shear moduli of 7–100 kPa (Wang et al., 2017) and bone osteoclasts face a Young’s moduli in the GPa range (Mirzaali et al., 2016). Moreover, pathological disorders such as tumors (Emon et al., 2018) or tissue fibrosis (Wells, 2013) can also promote changes in the stiffness range these and other immune cells encounter.

It is becoming increasingly clear that macrophages respond to mechanical cues (McWhorter et al., 2013; Moshayedi et al., 2014; Ip and Medzhitov, 2015; Jain and Vogel, 2018; Li et al., 2018), but although considerable efforts have been made to understand how microenvironment stiffness influences macrophage phenotype and function, the data are inconclusive. Some studies suggest that stiffer substrates upregulate macrophage pro-inflammatory responses and polarize them towards an M1 phenotype (Blakney et al., 2012; Previtera and Sengupta, 2015; Okamoto et al., 2018; Hsieh et al., 2019; Sridharan et al., 2019), while others show that more compliant materials enhance their pro-inflammatory behavior (Adlerz et al., 2015; Scheraga et al., 2016; Gruber et al., 2018). Parameters such as the stiffness range, the adhesive ligand, the activation stimulus and the specific cell type used vary across the different studies and all these factors could influence the results. It is clear that further research is required to dissect how substrate stiffness modulates the behavior of macrophages and how this impacts on their ability to induce inflammatory responses.

Inflammasomes are one of the key elements required for the processing and release of the major pro-inflammatory cytokines IL-1β and IL-18 (Martinon et al., 2002; Dinarello, 2011; Kaplanski, 2018). They are multimeric complexes comprised by a characteristic pattern-recognition receptor (PRR) that acts as sensor protein, the adaptor protein apoptotic speck-like protein containing a caspase recruitment domain (ASC) and the effector pro-caspase-1. Different PRRs can assemble different inflammasomes in response to a variety of exogenous and endogenous danger signals (Malik and Kanneganti, 2017). Among them, the nucleotide oligomerization domain (NOD)-like receptor (NLR) family member NLRP3 reacts to several substances such as the K^+^ ionophore nigericin, extracellular adenosine triphosphate (ATP) or monosodium urate crystals (MSU) (Mariathasan et al., 2006; Martinon et al., 2006). For the NLRP3 inflammasome to become canonically active, two steps are required. First, a priming signal is necessary to activate the transcription factor NFκB. Several danger molecules containing pathogen-associated molecular patterns (PAMPs) and endogenous damage-associated molecular patterns (DAMPs) engage with specific PRRs (Takeuchi and Akira, 2010). These respond by activating downstream signaling pathways that trigger the activation of NFκB and its translocation into the nucleus, where it promotes the transcription and translation of cytokines such as TNF-α, IL-6, pro-IL-1β and pro-IL-18 (Dorrington and Fraser, 2019), and the synthesis of other inflammation-related proteins, including NLRP3 (Bauernfeind et al., 2009). After priming, a second danger stimulus triggers the oligomerization of NLRP3 and the recruitment of ASC and pro-caspase-1 to form an inflammasome (Agostini et al., 2004). Finally, this causes the activation of caspase-1, enzyme that cleaves pro-IL-1β and pro-IL-18 into their biologically active forms (Agostini et al., 2004) and simultaneously promotes pyroptotic cell death via cleavage and maturation of the pore-forming protein gasdermin D (GSDMD) (Liu et al., 2016). The induced pyroptosis enhances the secretion of cytokines and facilitates the release of intracellular danger molecules to potentiate the response of neighboring cells (Evavold et al., 2018).

Although extensive research has been done to understand which biochemical signals modulate inflammasome activation (Swanson et al., 2019), little is known about how mechanical cues could influence its formation. Recent work indicates that biophysical signals could also modulate inflammasomes (Ip and Medzhitov, 2015; Maruyama et al., 2018) and, importantly, it has been shown that mechanotransducers such as the cytoskeleton components F-actin and microtubules may play an important role as regulators of inflammasome assembly (Man et al., 2014; Burger et al., 2016; Magupalli et al., 2020). Despite that, whether substrate stiffness has an impact on macrophage inflammasome formation remains unknown.

Here, we use polyacrylamide hydrogels of varying elastic moduli to investigate how substrate stiffness modulates the pro-inflammatory response of macrophages and influences the activation of the NLRP3 inflammasome. Bone marrow-derived macrophages cultured on substrates with a Young’s modulus of 0.2 kPa adopted a radically different morphology from macrophages grown on 33.1 kPa polyacrylamide gels, exhibiting lower spreading area and higher circularity, with less extended processes but numerous ruffles and folds displayed on the cell surface. Despite not notably affecting most of their pro-inflammatory gene expression patterns, upon LPS priming and nigericin stimulation the more compliant hydrogels enhanced NLRP3 inflammasome assembly, pyroptosis onset and secretion of the cytokines IL-1β and IL-6. Limiting cell spreading through a micropatterning approach revealed that reduced cell area alone could not explain the enhanced inflammasome activation observed on lower substrate stiffness. In contrast, inhibiting myosin activity diminished the differences in inflammasome formation induced by varying substrate compliance. Together, these findings reveal the influence of substrate stiffness on the formation of the NLRP3 inflammasome and on its downstream effects and suggest a role of actomyosin contractility in mediating the integration of mechanical cues into the macrophage inflammatory machinery.

## Results

### Substrate Stiffness Modifies Macrophage Morphology

**FIGURE 1:**
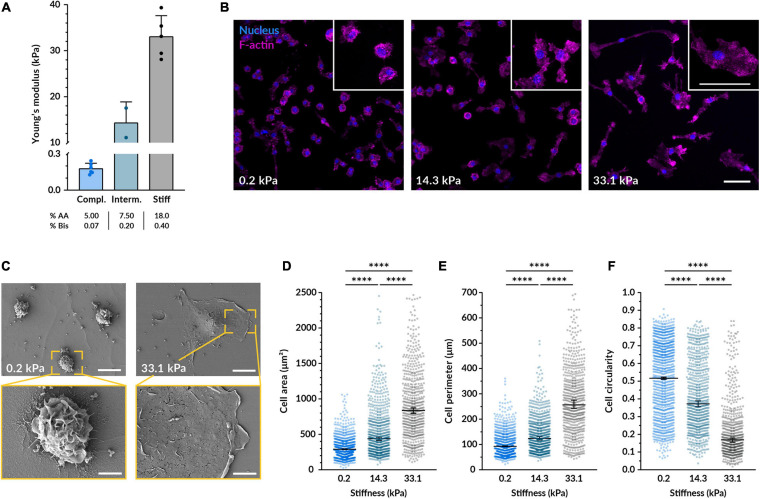
Substrate stiffness affects the morphology of macrophages. **(A)** Young’s moduli of the three different polyacrylamide 2D hydrogels used as cell substrate obtained by AFM. The mean ± SD was 0.18 ± 0.04 kPa for the compliant, 14.32 ± 4.54 kPa for the intermediate, and 33.07 ± 4.55 kPa for the stiff gel. **(B)** Representative confocal microscopy images of fluorescent BMDMs cultured on the different gels for 14–18 h. Nuclei shown in blue (DAPI) and F-actin in magenta (phalloidin-TRITC). Enlarged examples of cells of each condition are displayed in the insets. Scale bars, 50 μm. **(C)** Scanning electron micrographs of BMDMs on the compliant and stiff gels cultured as in **(B)**. Scale bars on top, 50 μm, and bottom, 20 μm. **(D–F)** Quantification of the cell area **(D)**, perimeter **(E)**, and circularity **(F)** from images taken under the same conditions as **(B)**. In all plots, each dot represents a cell and the black bars indicate mean ± SEM. The number of analyzed cells was 1117 for compliant, 771 for intermediate and 629 for stiff; data were obtained from 3 independent experiments done with cells from three different mice. Statistical analysis was performed using a Kruskal–Wallis ANOVA followed by Dunn’s *post hoc* analysis to obtain the multiple comparison *p*-values. ^****^p < 0.0001.

To investigate the effect of substrate stiffness on macrophages, we cultured murine bone marrow-derived macrophages (BMDMs) on flat polyacrylamide hydrogels of different rigidity but comparable adhesive RGD coating. We started testing three different stiffnesses with a mean Young’s moduli of 0.2, 14.3, and 33.1 kPa (Figure 1A). These stiffnesses encompass the different elastic moduli values found in the bone marrow, from 0.1 kPa in the central part and more vascularized areas to a range of 30–100 kPa near the bone surface (Ivanovska et al., 2017). After culturing BMDMs on the gels for 14–18 h, they were well adhered to the material in all conditions and their viability was above 85% (Figures 1B, 3E). In line with previously reported observations (Blakney et al., 2012; Patel et al., 2012; Adlerz et al., 2015; Hsieh et al., 2019; Sridharan et al., 2019; Xing et al., 2020), imaging F-actin revealed that while macrophages on more compliant polyacrylamide remained rounder and less spread, cells on stiffer substrates displayed a significantly larger area, longer processes and higher actin density in some clusters (Figures 1D–F). When quantifying their morphological features, we also determined that, compared to the most compliant gel (0.2 kPa), macrophages on the stiffest substrate (33.1 kPa) had a 229% higher mean spreading area, a 202% bigger mean perimeter and 57% lower mean circularity (Figures 1D–F). Therefore, the stiffer the substrate, the higher the spreading area and perimeter, and the lower the cell circularity. Out of the different hydrogel stiffnesses, for the remainder of the study we decided to use the most compliant (0.2 kPa) and the stiffest (33.1 kPa) materials because they were the most distant from each other and they consistently and substantially influenced macrophage morphology. To better observe whether the cells adopted different morphological features, we also acquired images of the macrophages on the hydrogels using scanning electron microscopy. Cells on 0.2 kPa gels had considerably more membrane ruffles and folds than cells on 33.1 kPa substrates, where the increased spreading was associated with a smoother plasma membrane (Figure 1C).

### Substrate Stiffness Influences Pro-Inflammatory Behavior of Macrophages

**FIGURE 2:**
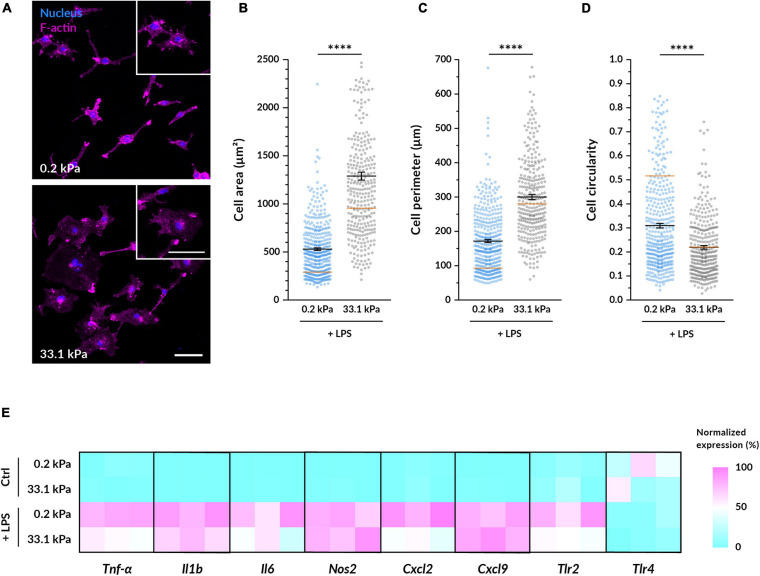
Substrate stiffness influences LPS-primed macrophages. **(A)** Representative fluorescence microscopy images of BMDMs cultured on the different gels for 14–18 h and then primed with 100 ng/ml LPS for 4.5 h. Nuclei are shown in blue and F-actin in magenta. Enlarged examples of cells of each condition are displayed in the insets. Scale bars, 50 μm. **(B–D)** Quantification of the cell area **(B)**, perimeter **(C)**, and circularity **(D)** from images taken under the same conditions as **(A)**. In all plots, each dot represents a cell, the black bars indicate mean ± SEM, and the reference orange bar indicates the mean of the non-primed macrophages in Figures 1D–F. The number of analyzed cells was 390 for compliant and 322 for stiff; and data were obtained from three independent experiments done with different animals. Statistical analysis was performed using a Mann–Whitney test. ^* * **^p < 0.0001. **(E)** Relative gene expression levels of pro-inflammatory genes in non-treated macrophages and in cells primed with 100 ng/ml of LPS for 6 h. Normalized data are presented as heatmaps (see Supplementary Figure S1 for absolute fold changes).

To study their pro-inflammatory response, we primed the BMDMs with lipopolysaccharide (LPS), a molecule present in the wall of gram-negative bacteria. LPS activates toll-like receptors (TLRs) present in the macrophage cell membrane and triggers downstream signaling cascades that promote the transcription of pro-inflammatory genes. We first assessed whether the morphological differences caused by varying substrate stiffness persisted after macrophage priming. After treatment with LPS for 4.5 h, we observed similar relative differences between the compliant and stiff conditions as in the non-primed macrophages (Figures 2A–D). Nevertheless, when compared to unstimulated, LPS-primed cells became larger on both substrates. On 0.2 kPa gels, LPS-treated macrophages had an 83% larger area, an 85% longer perimeter and a 40% lower circularity compared to untreated controls. On 33.1 kPa stiff substrates, they were only 38% more spread, had an 8% longer perimeter but similar circularity. The LPS-induced increase in spreading area and perimeter and the decrease in circularity were more notable in the cells cultured on the compliant than on the stiffer substrate. As previously reported (Jain and Vogel, 2018), LPS tends to promote cell spreading and, since macrophages were already well spread on the stiff gels before their priming, it seems logical that we detected more pronounced changes in the 0.2 kPa gels.

Next, we investigated whether substrate stiffness influences the expression levels of several pro-inflammatory genes after LPS priming via quantitative RT-PCR. In absence of LPS, the expression of the analyzed pro-inflammatory genes (*Tnf*-α, *Il6, Il1b, Nos2, Tlr2, Tlr4, Cxcl2*, and *Cxcl9*) was minimal except for *Tlr4* and there were no differences between macrophages cultured on 0.2 kPa and 33.1 kPa hydrogels (Figure 2E and Supplementary Figure S1). As expected, after LPS priming most of the gene expression levels were significantly increased and, interestingly, when comparing the two stiffnesses we detected a significantly higher expression higher expression of *Tnf*-α on the more compliant gels. Despite not being statistically significant, also the genes encoding for the cytokines *Il6* and *Il1b* were following a similar trend, and the receptor *Tlr2* and the chemokine *Cxcl2* were also upregulated on more compliant substrates. No differences in the expression of *Nos2*, *Tlr4*, and *Cxcl9* were detected.

**FIGURE 3:**
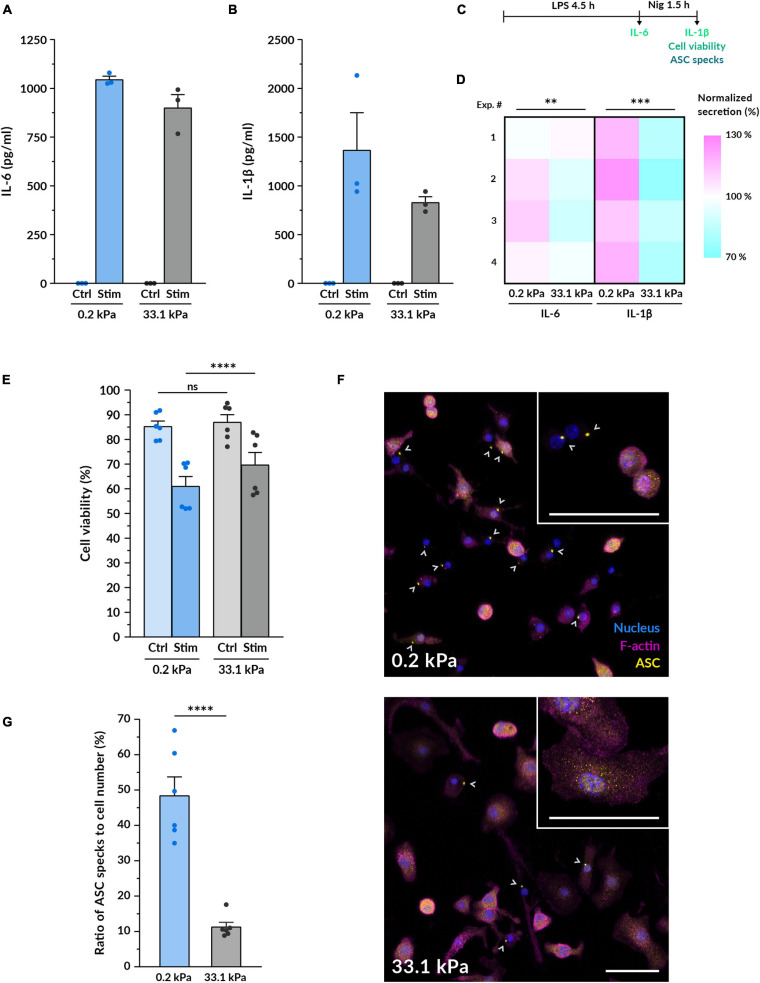
Macrophage pro-inflammatory response is upregulated by more compliant substrates. **(A,B)** Quantification of the protein levels of the cytokines IL-6 **(A)** and IL-1β **(B)** secreted by macrophages on compliant and stiff polyacrylamide hydrogels via ELISA. BMDMs were cultured for 14–18 h on the hydrogels, primed with 100 ng/ml LPS for 4.5 h and stimulated with 10 μM nigericin for 1.5 h. IL-6 supernatants were collected after LPS priming, for IL-1β after nigericin treatment. Results of one representative experiment are shown (mean ± SEM), each dot represents one replicate. All experiments were independently repeated three times using cells from three different mice and with similar results. **(C)** Scheme of the experimental treatment applied in **(A–G)**. **(D)** Heatmap of the IL-6 and IL-1β cytokine quantification performed as described in **(A,B)**. Each square represents an independent experiment. Statistical analysis was performed using a 1D linear mixed model and *p*-values were determined by a likelihood ratio test. ^∗∗∗^*p* < 0.001. **(E)** Assessment of cell viability after macrophage priming and stimulation. Cell viability was determined by LDH assay for untreated control BMDMs cultured on gels and for macrophages treated as described in **(A,B)**. Mean ± SEM are shown and each dot represents a replicate obtained from three independent experiments. Statistical analysis was performed using a 1D linear mixed model and *p*-values were determined by a likelihood ratio test. ^∗∗∗^*p* < 0.001. **(F)** Representative confocal microscopy images of fluorescent ASC specks as a measure of inflammasome formation. BMDMs cultured on the different gels for 14–18 h, primed and stimulated as in **(A,B)**. Nuclei shown in blue, F-actin in magenta and the inflammasome linker protein ASC in yellow. Gray arrowheads indicate ASC specks, markers of inflammasome assembly. Insets display higher magnification examples of cells on each stiffness. Scale bars, 50 μm. **(G)** Quantification of the ratio of ASC specks to cell number. Mean ± SEM are shown and each dot indicates one of the different replicates obtained from three independent experiments. The total number of cells analyzed was 4327 (compliant hydrogels) and 5030 (stiff hydrogels). Statistical analysis was performed using a 1D linear mixed model and *p*-values were determined by a likelihood ratio test. ns, not significant, **p < 0.01, ***p < 0.001, ****p < 0.0001.

While substrate stiffness did not strongly alter pro-inflammatory gene expression, we decided to assess whether it influenced the synthesis and release of the cytokines IL-6 and IL-1β. Priming with LPS alone is sufficient to induce the production and secretion of IL-6, so we quantified its concentration after 4.5 h of treatment. When macrophages were cultured on compliant gels, we detected on average an 11% higher concentration of secreted IL-6 in the supernatant (Figures 3A,C,D). To secrete IL-1β, macrophages need to be not only primed with LPS but also provided with a second stimulus that triggers the final maturation and release of the cytokine. In this study, we employed nigericin: a potent K^+^ efflux inducer that triggers the formation of the NLRP3 inflammasome. This large multiprotein complex mediates the final processing of IL-1β, which is then secreted outside the cell (Martinon et al., 2002; Agostini et al., 2004). Following the same trend as with IL-6, we found that after 1.5 h nigericin stimulation macrophages on 0.2 kPa hydrogels consistently secreted higher amounts of IL-1β, representing a mean 44% increase over macrophages on 33.1 kPa hydrogels (Figures 3B–D).

Since nigericin treatment also triggered the onset of pyroptotic cell death, we expected that the higher IL-1β release would be associated with increased cell death. For this reason, we also assessed cell viability by measuring LDH release (Figures 3C,E and Supplementary Figure S2). Before macrophage priming and activation, we did not find a significant difference between both stiffnesses. After 90 min of incubation with nigericin, however, we detected that macrophage viability was significantly lower when stimulated while being cultured on the 0.2 kPa polyacrylamide, indicating that pyroptosis was increased on the compliant gels (Figures 3C,E). While this difference was also detectable after 60 min and 6 h of initiating the nigericin treatment (Supplementary Figure S2), we kept using the 90 min timepoint for the rest of the study because at this point the macrophage pyroptotic response was robust enough but still submaximal. Collectively, these data show that substrate stiffness influences pro-inflammatory cytokine secretion and suggest that the upstream signaling events leading to cytokine maturation and release may become enhanced when macrophages are cultured on a more compliant substrate.

According to this hypothesis, we tested whether substrate stiffness influenced the formation of the NLRP3 inflammasome, which is the corresponding inflammasome activated by the nigericin-induced K^+^ efflux. We imaged macrophage inflammasomes by immunostaining for the linker protein ASC after the same stimulation treatment. ASC is the protein linking NLRP3 and pro-caspase1, three proteins that, upon K^+^ efflux, cluster together forming a 1-μm structure that can be easily identified in fluorescence images (Figure 3F). Cells that have an assembled inflammasome and triggered the onset of pyroptosis tend to progressively reduce their cell area, which we could especially distinguish on the better spread cells on stiff gels (Supplementary Figure S3). Besides ASC-positive specks, we also observed some decreased levels of F-actin, indicating the impairment of the actin cytoskeleton caused by the activation of the pyroptotic machinery. By determining the ratio of ASC-positive specks to cell number after 90 min of nigericin stimulation, we found that macrophages on 0.2 kPa gels displayed 4 times more ASC specks than macrophages on 33.1 kPa (Figure 3G). This strongly indicates that the assembly of the NLRP3 inflammasome is upregulated in cells on more compliant substrates. Altogether, our data show that more compliant substrates enhance the assembly of macrophage NLRP3 inflammasomes, the secretion of the pro-inflammatory cytokines IL-1β and IL-6 and the release of the pyroptotic marker LDH, suggesting that substrate stiffness may modulate the formation of the inflammasome and the downstream activation of the pyroptotic machinery.

### Inhibiting Myosin but Not Limiting Cell Spreading Suppresses Stiffness-Induced Differences in Inflammasome Formation

**FIGURE 4:**
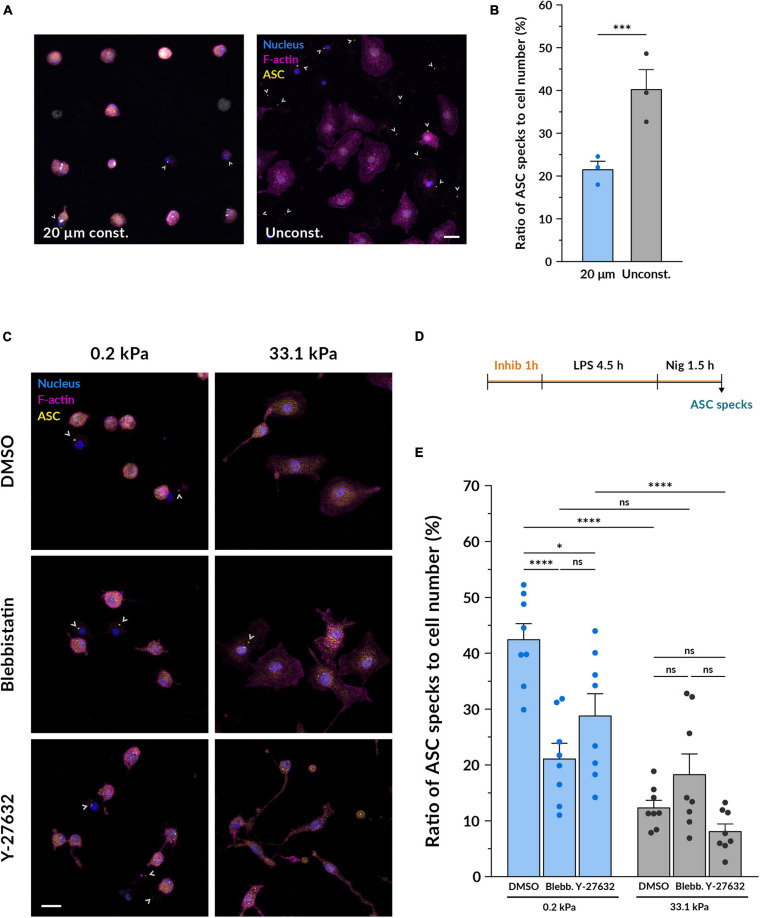
Inhibiting myosin but not limiting cell spreading diminishes the differences induced by stiffness on inflammasome formation. **(A)** Representative confocal microscopy images of inflammasome formation under cell confinement. BMDMs were cultured on fibronectin-coated circular islands micropatterned on a glass substrate with a diameter of 20 μm. Unconfined cells were grown on fibronectin-coated glass coverslips growing in an unconfined manner. After 14–18 h of culture, they were primed with 100 ng/ml LPS for 4.5 h and stimulated with 10 μM nigericin for 1.5 h. Nuclei shown in blue, F-actin in magenta and ASC in yellow. Scale bar, 20 μm. **(B)** Quantification of the ratio of ASC specks to cell number. Mean ± SEM are shown and each dot indicates an independent experiment done with BMDMs from different mice. The total number of cells analyzed was 1560 for the 20 μm confined and 1524 for the unconfined adhesion area. Statistical analysis was performed using a 1D linear mixed model and *p*-values were determined by a likelihood ratio test. ^* * **^p < 0.0001. **(C)** Representative confocal microscopy images of inflammasome formation under actomyosin inhibition. BMDMs cultured on hydrogels for 14–18 h were pretreated with either 1:1700 DMSO as a control, 10 μM blebbistatin or 10 μM Y-27632 for 1 h. Keeping the respective inhibitor molecules, cells were then primed with 100 ng/ml LPS for 4.5 h and stimulated with 10 μM nigericin for 1.5 h. Nuclei shown in blue, F-actin in magenta and ASC in yellow. Scale bar, 20 μm. **(D)** Scheme of the experimental treatment applied in **(C,E). (E)** Quantification of the ratio of ASC specks to cell number. Mean ± SEM are shown and each dot indicates one of the different replicates obtained from three independent experiments. The total number of cells analyzed was, for compliant gels, 4673 (DMSO), 3528 (blebbistatin) and 5182 (Y-27632); and for stiff gels, 4859 (DMSO), 5447 (blebbistatin), and 5214 (Y-27632). Statistical analysis was performed using a one-way ANOVA followed by Bonferroni’s *post hoc* analysis to obtain the multiple comparison *p*-values. ns, not significant, *p < 0.05, ***p < 0.001, ****p < 0.0001.

We first hypothesized that the distinct cell spreading we had observed on compliant and stiff gels could serve as a mechanosensory element directly involved in modulating the inflammatory response of macrophages. To test if spreading alone affects inflammasome formation, we analyzed as above ASC specks, this time limiting the macrophage spreading area. Therefore, we cultured BMDMs on fibronectin-coated round islands with a diameter of 20 μm micropatterned on a glass substrate (Figure 4A). The adhesion area of each island was 314 μm^2^, resembling the mean spreading area of macrophages on compliant hydrogels (Figure 1D). Confined macrophages were compared to unconfined cells cultured on the same glass substrate functionalized with non-micropatterned fibronectin (Figure 4A). Surprisingly, after nigericin stimulation confined macrophages had on average significantly less ASC specks than macrophages grown in an unconfined area (Figure 4B). Limiting cell spreading under these conditions did not recapitulate what we had observed on the compliant hydrogels since it did not enhance inflammasome formation but actually reduced it. Therefore, cell spreading alone does not seem to be the element positively modulating the increased response of macrophages on softer substrates.

Given that the conformation of the F-actin cytoskeleton in BMDMs is also altered by substrate stiffness and actomyosin is involved in mechanosensing (Elosegui-Artola et al., 2018), we decided to investigate whether actomyosin contractility could play a role as a mechanotransducer in the context of macrophage pro-inflammatory activation. To this end, we primed and stimulated macrophages under the effect of blebbistatin, a non-muscle myosin II inhibitor, and Y-27632, which blocks the activity of ROCK1 and ROCK2 (Figures 4C,D). Compared to the DMSO control, cells incubated with 10 μM blebbistatin did not show notable changes in their morphology (Figure 4C and Supplementary Figure S4), as also noted in Jain and Vogel (2018). Nevertheless, when using 10 μM Y-27632, macrophages qualitatively appeared thinner and formed some elongated processes, a change that could be especially perceived when imaging the cells treated with nigericin on stiff substrates. After quantifying inflammasome formation we first observed that when growing macrophages on 0.2 kPa gels, both blebbistatin and Y-27632 reduced the ratio of ASC specks (Figure 4E). On the other side, when cultured on 33.1 kPa substrates there were no differences to the DMSO control. When comparing the data of the two substrate stiffnesses under the effect of the each inhibitor, it was particularly interesting to note that blebbistatin greatly diminished the differences between the compliant and stiff gels. In contrast, inhibiting ROCK did not seem to balance out the differences induced by culturing macrophages on substrates of different stiffness. These data indicate that inhibiting actomyosin contractility with blebbistatin had a significant impact on the enhanced ability of macrophages to assemble the NLRP3 inflammasome on more compliant substrates, decreasing the inflammasome formation rate and equalizing it to the one on stiffer gels. Altogether, these results suggest that actomyosin contractility may be an element involved in the integration of substrate stiffness as a modulatory parameter of macrophage inflammasome formation.

## Discussion

It is becoming increasingly clear that macrophages respond to mechanical cues and that biophysical stimuli such as interstitial fluid flow (Li et al., 2018), hyperosmotic shocks (Ip and Medzhitov, 2015) or the modification of cell shape (McWhorter et al., 2013) can have an impact on their phenotype and function. In the present study, we explored the mechanosensitive response of macrophages by exposing BMDMs to substrate stiffness in the range of 0.2 to 33.1 kPa. Our results indicate that more compliant substrates increase the sensitivity of BMDMs to pro-inflammatory stimuli, enhancing inflammasome formation, pyroptosis onset and cytokine release, and that this might be mediated through actomyosin contractility.

The culture of macrophages on substrates of varying stiffness affected several morphological parameters including spreading area, circularity and membrane topography. This is in line with what most previous studies have shown in several stiffness ranges and different macrophage cell types, including murine BMDMs (Hsieh et al., 2019), RAW 264.7 murine cells (Blakney et al., 2012; Patel et al., 2012), THP-1-differentiated macrophages (Sridharan et al., 2019; Xing et al., 2020) and human monocyte-derived macrophages (Adlerz et al., 2015). We report that BMDMs on 0.2 kPa polyacrylamide substrates upregulated their pro-inflammatory response when compared to the 33.1 kPa hydrogels. Despite not observing major changes in the expression of pro-inflammatory genes, upon LPS priming and nigericin stimulation we detected higher secretion levels of IL-6 and IL-1β on the most compliant hydrogels. Several studies using polyacrylamide gels within a similar stiffness range also reported an enhanced secretion of pro-inflammatory cytokines on more compliant substrates. For instance, Scheraga et al. (2016) cultured BMDMs on fibronectin-coated polyacrylamide gels with a Young’s modulus between 1 and 25 kPa. Upon LPS priming, they detected higher concentrations of IL-1β on their most compliant gels and, interestingly, they detected higher levels of the anti-inflammatory cytokine IL-10 on the stiffer material. Using the same type of gels, cells, and stimulus, Gruber et al. (2018) reported that BMDMs released higher amounts of the pro-inflammatory cytokine TNF-α on 1 kPa substrates than on 20 and 150 kPa hydrogels. In this case, though, the concentration of IL-10 was higher on the compliant substrates. In line with that, Patel et al. (2012) cultured RAW 264.7 macrophages on poly-D-lysine (PDL)-coated polyacrylamide gels between 1 and 150 kPa and quantified that the lower the stiffness, the higher the secretion of the TNF-α upon LPS challenge. Additionally, some recent reports point out that higher stiffness could promote a shift from M1 to M2-like phenotype. Carnicer-Lombarte et al. (2019) tested the influence of different PDL-coated polyacrylamide gels in the phenotype of BMDMs, detecting higher levels of M1-like genes on 1 kPa gels and higher levels of M2-like genes on 50 kPa materials. And recently, Xing et al. (2020) described that when THP-1 macrophages were cultured on collagen-coated PAA hydrogels between 6 and 16 kPa, the stiffer substrates promote higher expression of M2 markers.

In contrast, there are other reports stating that stiffer microenvironments are the ones upregulating macrophage pro-inflammatory behavior (Blakney et al., 2012; Previtera and Sengupta, 2015; Okamoto et al., 2018; Hsieh et al., 2019; Sridharan et al., 2019). For example, Previtera and Sengupta (2015) exposed BMDMs to polyacrylamide gels ranging from 0.3 to 230 kPa and after LPS treatment they detected higher concentrations of secreted IL-1β, TNF-α and NO on stiffer substrates. This discrepancy in the response of macrophages across different studies might be caused by the choice of different cell lines, adhesion protein, its coating density or the type of stimulatory challenge. In the present study we should note that we collected the samples to quantify IL-6 only after 4.5 h of LPS priming and, unlike all the mentioned studies, we induced the release of IL-1β with the ionophore nigericin. Moreover, it is important to consider the possibility that macrophages might not react to substrates of varying stiffness in a monotonous manner. Sridharan et al. (2019) observed that in THP-1-derived macrophages the influence of material stiffness on phagocytosis and several M1 genes and cytokines followed a biphasic response. The data obtained by Gruber et al. (2018) in RAW 264.7 cells reported a similar behavior in LPS-induced TNF-α secretion. The biphasic response to substrate mechanics has already been described in other cell types such as fibroblasts (Wang et al., 2019) and, therefore, the existence of a non-linear relationship between substrate stiffness and macrophage behavior should not be discarded until more data is collected.

The inflammasome represents one of the main signaling hubs activated in macrophages upon inflammatory stimuli. Despite the fact that most of the described inflammasome regulators consist of biochemical signals (Swanson et al., 2019), recent studies suggest that mechanical cues could also be modulating its formation. For example, cycling stretch has been shown to downregulate the release of IL-1β by inhibiting NLRP3 inflammasome formation (Maruyama et al., 2018) and hyperosmotic shocks have been described to trigger the reverse effect (Ip and Medzhitov, 2015). Here, we provide evidence for the first time that microenvironment stiffness can also influence the activation of the NLRP3 inflammasome. In order to test whether the higher IL-1β secretion we observed on more compliant gels was accompanied by an upregulation of inflammasome assembly, we quantified the presence of ASC specks as a proxy of inflammasome activation. Our results showed that, upon LPS priming and nigericin stimulation, the most compliant substrates led to increased inflammasome formation. This suggests that substrate stiffness plays a role in regulating the assembly of the NLRP3 inflammasome and indirectly influences the activity of pyroptotic effectors such as caspase-1 and gasdermin D, which would explain the higher amounts of released LDH we also detected in the supernatant. All these effects could be caused by a decrease in the inflammasome activation threshold or by higher formation rates, but more detailed time-dependent data is necessary to understand the specific dynamics behind this process.

Based on the severe differences in cell shape and membrane topography induced by the compliant and stiff gels, we first tried to assess whether there was a relationship between macrophage spreading and inflammasome assembly. Larger cell spreading could indirectly cause an increase in membrane curvature and tension which mechanosensory elements like caveolae and mechanically gated ion channels could detect (Le Roux et al., 2019). Here, we firstly hypothesized that the lower spreading BMDMs adopted on more compliant substrates might have enhanced the formation of the NLRP3 inflammasome. We found that, however, limiting cell spreading on a stiff substrate did not enhance inflammasome formation but rather reduced it. Despite the difference that this experiment was not directly performed using stiff polyacrylamide gels but stiff glass substrates, these imply that reducing cell spreading alone may not recapitulate the downstream inflammatory effects we observed on gels of different stiffness. Previous research done by Jain and Vogel (2018) showed that spatial confinement of BMDMs on a glass surface downregulated the expression of pro-inflammatory genes *Il6*, *Cxcl9*, *Il1b* and *Nos2* and the subsequent secretion of TNF-α, IL-6 and IL-12 via the indirect modulation of chromatin compaction. Our data support the idea that cell confinement reduces the response of macrophages to pro-inflammatory stimuli and suggests that the NLRP3 inflammasome could be an additional signaling component affected by it.

Actomyosin contractility has been described as one of the key elements of the stiffness mechanotransduction machinery (Elosegui-Artola et al., 2018) and as necessary for macrophages to exert traction forces on their substrates (Hind et al., 2015). Here we tested whether interfering with it would have any effect on the activation of the NLRP3 inflammasome in BMDMs exposed to different material stiffness. The inhibition of myosin II’s motor activity with blebbistatin during macrophage priming and stimulation strongly decreased the amount of inflammasome-activated cells on the compliant hydrogels and slightly increased it on the stiff ones, reducing the original differences between both stiffnesses. Inhibiting ROCK1 and ROCK2 with Y-27632 also downregulated the formation of inflammasomes on compliant gels but in a less substantial manner than blocking myosin II activity alone. We should note that while blebbistatin specifically inhibits the motor activity of myosin II, pharmacologically blocking ROCK1/2 with Y-27632 not only inhibits the phosphorylation of myosin but also promotes F-actin destabilization (Maekawa et al., 1999; Horváth et al., 2020), possibly contributing to the different results obtained with these two molecules. Moreover, as shown in Hind et al. (2016), the generation of traction forces in M1 macrophages depends on myosin II activity but not on upstream ROCK activation. As they propose, perhaps other myosin II regulatory proteins such as MLCK contribute to promote actin contractility, explaining why we did not observe an effect of the same magnitude as when directly inhibiting myosin II. We speculate that there might be an optimal level of actomyosin contractility at which inflammasome formation is promoted and that macrophages on more compliant substrates might be closer to it, leading to an increase in inflammasome assembly. Overall, our results suggest that actomyosin contractility may play an important role in the integration of mechanical signals into the NLRP3 inflammasome regulatory pathways, but further studies are required to determine the exact sequence of events needed to couple them.

Recent papers also propose the idea that the actin cytoskeleton may be involved directly in the control of inflammasome activation. Man et al. (2014) showed that, upon exposing macrophages to *Salmonella*, actin polymerization is necessary for NLRC4 inflammasome formation, pyroptosis onset and IL-1β release, and Burger et al. (2016) determined that F-actin interacts with NLRP3 and ASC and negatively regulates the activity of the NLRP3 inflammasome. Moreover, other cytoskeletal components have also been described as regulators of inflammasome activation. dos Santos et al. (2015) detected that the intermediate filament vimentin interacts with NLRP3 and regulates inflammasome formation and Magupalli et al. (2020) showed that NLRP3 and pyrin inflammasomes are assembled at the centrosomes with the requirement of HDAC6-dependent microtubule transport. Since it has been reported that the actin cytoskeleton can cross-talk with microtubules to guide and redirect their growth (López et al., 2014), we could speculate that perhaps certain levels of actomyosin contractility could be required to enable a correct organization of microtubules, which would then facilitate inflammasome formation. The importance the cytoskeleton may have as an additional inflammasome regulator has just begun to be uncovered and what the exact link between mechanical cues, cytoskeleton activity and inflammasome formation is remains an open question to be addressed in the future.

The study of immune mechanosensing offers the possibility to provide a better insight of pathologies that trigger changes in the mechanical properties of affected tissues. For instance, the downregulation of the pro-inflammatory response of macrophages on substrates with higher stiffness could be relevant in the context of cancer. Tumor-associated macrophages (TAMs) are one of the key immune cell types present in the tumor microenvironment. TAMs with an M2-like phenotype infiltrated within cancerous tissues secrete cytokines that downregulate the immune response against neoplastic cells and they tend to favor tumor growth (Zhou, 2020). Compared to their healthy tissue counterparts, primary tumors are often associated with higher tissue stiffness (Nia et al., 2020). For example, in murine breast cancer the elastic modulus increases from an average of 0.17 kPa in a normal mammary gland to 4 kPa in mammary tumors (Paszek et al., 2005). And in human brain tumors the elastic modulus can increase from a few hundred Pa in non-cancerous tissue up to 13.5 kPa in advanced glioblastomas (Miroshnikova et al., 2016). Thus, in synergy with biochemical signals, the increased stiffness of malignant tissues may promote downregulation of an M1-like pro-inflammatory response of TAMs and induce the polarization towards an M2-like phenotype, promoting cancer progression. Additional knowledge on how microenvironment stiffness governs macrophage phenotype and function could, thus, help identifying novel therapeutic targets. Moreover, a deeper understanding of macrophage mechanobiology would also be beneficial for the development of better immunomodulatory treatments and the design of superior biomaterials to be used in implantable medical devices (Lacour et al., 2016).

## Materials and Methods

### Preparation of Polyacrylamide Hydrogels (PAA Gels)

Gels were produced on aminosilanized glass coverslips. Briefly, 13 mm round glass coverslips were washed with 0.1 M NaOH for 15 min, washed with ethanol and water and dried. They were incubated for 20 min in a solution of 0.1% (v/v) allyltrichlorosilane and 0.1% (v/v) triethylamine diluted in chloroform, washed and dried. Finally, they were covered with 0.5% glutaraldehyde for 30 min, washed and dried. To obtain hydrogels with a range of different stiffness, acrylamide (AA) and N,N′-methylenebisacrylamide (BisA) were pre-mixed in different proportions. For compliant gels we used 5% AA and 0.07% BisA; for intermediate 12% AA and 0.2% BisA; and for stiff 18% AA and 0.4% BisA (all v/v and dissolved in PBS). Tetramethylethylenediamine (TEMED) was added to the pre-mixes to a final concentration of 0.3% (v/v). The mixture was degassed for 10 min. We used custom-made methylsulfone acrylate (MS) monomers as a thiol-reactive compound for functionalization with adhesion molecules, as recently described in Farrukh et al., 2016. The MS monomers are incorporated to the AA/BisA mix and after the gels are polymerized they can react with the thiol group present at the Cys residue of the employed adhesion peptide. This strategy enables the uncoupling of polyacrylamide stiffness from adhesion ligand density, ensuring a comparable density of peptides between different hydrogels. MS monomers were dissolved at 32 mg/ml in dimethylformamide (DMF). To initiate the polymerization, a final mixture 1:8 (v/v) of MS monomers and the AA/BisA pre-mixes was prepared and ammonium persulfate (APS) was added to a final concentration of 0.1% (w/v). 9.3 μl of the solution were placed between a glass coverslip and a flexible hydrophobic polyester sheet to gel for 30 min. Polymerized hydrogels were peeled off, immersed in water in a 24-well plate to swell for a minimum of 1 h, washed, UV-sterilized for 30 min and washed again. Hydrogels were functionalized with 0.5 mg/ml of cRGD-Phe-Cys (Pepnet #PCI-3686-PI) diluted in ddH_2_O at room temperature overnight. They were finally washed and then stored in water at 4°C for a maximum of 14 days until they were used for either mechanical characterization or cell culture. All chemicals mentioned were from Sigma-Aldrich unless specified.

### Mechanical Characterization of PAA Hydrogels by Atomic Force Microscopy (AFM)

Hydrogels bound to the glass coverslips were mounted on a 35 mm dish using vacuum grease and covered with PBS at room temperature. The characterization was performed with a Nanowizard 4 (JPK Instruments) using cantilevers (arrow T1, Nanoworld) equipped with 5 μm diameter polystyrene beads (microParticles GmbH) and calibrated with the thermal noise method. Gels were probed in liquid with an indentation speed of 5 μm/s and a relative force setpoint ranging from 0.6 to 8 nN to achieve comparable indentation depths of approximately 2 μm. The obtained force-distance curves were analyzed using the JPK data processing software. Parts of the curves corresponding to the first 2 μm of indentation depth were fitted using the Hertz/Sneddon model for a spherical indenter and Poisson ratio of 0.5 was assumed (Hertz, 1881; Sneddon, 1965).

### Macrophage Cell Culture, Stimulation, and Inhibitors

Primary bone marrow-derived macrophages (BMDMs) were produced by cultivating bone marrow harvested from C57BL/6J young mice (Janvier Labs; ethics approval number DD24.1-5131/396/9, Landesdirektion Sachsen) in BMDM medium consisting of high glucose DMEM + GlutaMAX (Thermo Fisher Scientific), 10% heat-inactivated FBS (v/v; Thermo Fisher Scientific), 1% penicillin-streptomycin (v/v; Thermo Fisher Scientific) and 20% L929 conditioned media (v/v) on Corning^TM^ not TC-treated petri dishes (Sigma-Aldrich) for 6 days. Differentiated BMDMs were detached, seeded on hydrogels within a 24-well plate format at the concentration specified for each experimental approach and cultured for 14–18 h. For LPS priming, cells were challenged with 100 ng/ml ultrapure LPS from *Escherichia coli* (InvivoGen) for 4.5 h for most of the experiments and for 6 h for the gene expression experiments. For nigericin stimulation, BMDMs were treated with 10 μM nigericin (InvivoGen) for 1.5 h. For inhibitor experiments, 0.06% DMSO (v/v; Sigma-Aldrich), 10 μM blebbistatin (Sigma-Adlrich #B0560) or 10 μM Y-27632 (Sigma-Aldrich #Y0503) were used. BMDMs were pre-treated with the inhibitors for 1 h, and the inhibitors were kept in the medium during the subsequent priming with LPS priming and stimulation with nigericin.

### Fluorescence Confocal Microscopy and Image Analysis

Cells were cultured at a density of approximately 50000 cells/cm^2^ (10^5^ in a 24-well plate). They were fixed with 4% paraformaldehyde (v/v; Thermo Fisher Scientific) for 12 min and permeabilized with 0.2% Triton^TM^ X-100 (v/v; Sigma-Aldrich) for 10 min. Blocking was done with 10% goat serum (v/v; Jackson ImmunoResearch), 0.1% bovine serum albumin (w/v; Sigma-Aldrich) for 1 h. To visualize ASC specks, samples were incubated with anti-ASC antibody (1:400; pAb AL177, AdipoGen #AG-25B-0006) overnight at 4°C and Alexa Fluor 488 anti-rabbit (1:400; Thermo Fisher Scientific #A-11034) for 1 h at room temperature. Nuclei and F-actin were stained with DAPI (1:2000; Sigma-Aldrich #32670) and phalloidin-TRITC (1:800; Sigma-Aldrich #P1951), respectively, for 1 h. All washes were done with PBS. Samples were mounted by inverting them over a #1.5 glass coverslip with a PBS drop to avoid excessive drying.

Cells were imaged using an LSM700 inverted confocal microscope and a 20x/0.8 objective (Zeiss), acquiring z-stacks with 0.89 μm steps in each position of interest. Image analysis was done using the software Image J/Fiji (Schindelin et al., 2012; Schneider et al., 2012) and Ilastik (Berg et al., 2019). Cell nuclei and cell body were automatically segmented based on the intensity signal of the DNA and the F-actin labeling, respectively, and morphological parameters were extracted. Circularity was calculated as $4\,\pi \,\frac{A\,r\,e\,a}{P\,e\,r\,i\,m\,e\,t\,e\,r^{2}}$. The ratio of specks to cell number was determined semi-automatically. Briefly, ASC specks were quantified manually, considering only mature, clearly formed single specks, with an approx. diameter of 1 μm. Since pyroptosis causes the release of cytoplasmic material out of the cell, both intra- and extracellular specks were included in the analysis. Macrophages with more than one mature speck were highly rare, counted as single-specked and included into the analysis. The number of cells per image was obtained by automatically segmenting their nuclei.

### Scanning Electron Microscopy (SEM)

Samples were fixed in 1 % glutaraldehyde in 100 mM phosphate buffer for at least 2 hours at room temperature and then washed in buffer (2x) and in water (4x). Samples were postfixed in 1% osmium tetroxide in water, washed several times in water, dehydrated in ascending ethanol concentrations (30, 50, 70, 90, 96% ethanol, 3x 100% ethanol on molecular sieve), and critical-point-dried using the Leica CPD300 drier (Leica Microsystems). Samples were mounted on 12 mm aluminum stubs, sputtered with gold (60 mA, 60 sec), and analyzed with a Jeol JSM 7500F cold field emission scanning electron microscope (Jeol Germany GmbH; acceleration voltage: 5 kV, emission: 10 μA, working distance: 8 mm, detector: lower secondary electron detector).

### Gene Expression Analysis Using Quantitative Real-Time PCR (qRT-PCR)

Total RNA was extracted from 1.2 × 10^6^ BMDMs grown oncompliant/stiff gels using the RNeasy Mini Kit (Qiagen #74104). Forthis, 6 wells with 2 × 10^5^ cells each were pooled for every experimental condition. For each stiffness, 6 wells were primed with lipopolysaccharide, while 6 wells received no treatment. Reverse transcription of 1 μg RNA was performed with iScript^TM^ Advanced cDNA Synthesis Kit (Bio-Rad #1725037), using a combination of oligo(dT) and random hexamer primers. qRT-PCR was performed at 56°C using GoTaq qPCR Mastermix (Promega #6002) on a Stratagene cycler Mx3005P system. Several primers of pro-inflammatory genes were used (Supplementary Table S1). Samples were run in duplicates and expression levels were normalized to the geometric mean of β-*actin*, *Gapdh*, and *18S* rRNA controls. Relative expression values were calculated as 2^(–ΔΔCT)^ (relative to geometric mean of housekeeping genes and plastic controls). Fold-changes can be found in Supplementary Figure S1. For heatmaps, relative expression values were normalized to values between 0 and 100% for each gene. Heatmaps were generated in R (R Core Team, 2020).

### Cytokine Quantification Assays

Bone marrow-derived macrophages were cultured at a density of approximately 100000 cells/cm^2^ (2⋅10^5^ in a 24-well plate). Supernatants from cell cultures were collected and dead cells removed by centrifugation. The amounts of IL-6 and IL-1β were determined using the IL-6 Mouse Uncoated ELISA Kit (Thermo Fisher Scientific #88-7064-88) and the IL-1β Mouse Uncoated ELISA Kit (Thermo Fisher Scientific #88-7013-88), respectively, according to the manufacturer’s instructions. Final absorbance was measured with a TECAN Infinite Pro plate reader, subtracting the 570 nm values from the 450 nm.

### Cell Viability Assay

Bone marrow-derived macrophages were also seeded at density of approximately 100000 cells/cm^2^ (2⋅10^5^ in a 24-well plate). Lactate dehydrogenase (LDH) activity in supernatants was measured using the LDH-Glo^TM^ Cytotoxicity assay (Promega #J2380) according to the manufacturer’s instructions. Luminescence was recorded with a GloMax^TM^ 96 microplate luminometer (Promega). Total amount of cells was inferred by lysing cells with Triton^TM^ X-100 at the end of the experiments and comparing the values to the ones of an included standard curve.

### Cell Micropatterning

Micropatterned glass slides were purchased from 4Dcell. The glass slides consisted of different areas with adhesion disks of a specific diameter ranging from 10 to 100 μm and non-adherent surface was passivated with PEG. The micropatterned surface was functionalized using 0.1 mg/ml fibronectin and cells were seeded at a density of approximately 10400 cells/cm^2^ (10^5^ in a 6-well plate). Cells were incubated overnight and the next day unattached cells were washed out before the start of the experiment.

### Statistical Analysis

Statistical tests are indicated in each plot. Linear mixed model analysis was performed using R (R Core Team, 2020) and the rest of statistical analysis was performed using GraphPad Prism 6 (GraphPad Software). All data are presented as mean ± SEM unless specified. In all cases, *p* values < 0.05 were considered statistically significant (ns, not significant; ^∗^*p* < 0.05; ^∗∗^*p* < 0.01; ^∗∗∗^*p* < 0.001; ^****^p < 0.0001).

## Data Availability Statement

The raw data supporting the conclusions of this article will be made available by the authors, without undue reservation.

## Ethics Statement

The animal study was reviewed and approved by DD24.1-5131/396/9.

## Author Contributions

JG, CB, AT, and JCE conceived the project and designed the experiments. JCE carried out bulk of the experiments. AF and AC synthetized and provided MS monomers for the polyacrylamide gels. SA and AT optimized the hydrogel preparation. CS performed qRT-PCR experiments. JCE, CS, SA, and AT analyzed the data and performed its evaluation. JCE, AT, JG, and CB wrote the manuscript. All authors contributed to the article and approved the submitted version.

## Conflict of Interest

The authors declare that the research was conducted in the absence of any commercial or financial relationships that could be construed as a potential conflict of interest.
