# Supplementary material for: Compliant Substrates Enhance Macrophage Cytokine Release and NLRP3 Inflammasome Formation During Their Pro-Inflammatory Response
Source: Front Cell Dev Biol. 2021 Mar 29;9:639815. doi: 10.3389/fcell.2021.639815 (PMC8039395; doi:10.3389/fcell.2021.639815)
Supplement: Supplementary file 1 [file Table_1.docx]

Supplementary Material

**Supplementary Table 1.** Primers used for the qRT-PCR experiments.

| **Oligo name** | **Primer sequence (5' --> 3') FWD** | **Primer sequence (5' --> 3') REV** | **Species** |
| --- | --- | --- | --- |
| **mACTB** | GATCAAGATCATTGCTCCTCCTG | CGCAGCTCAGTAACAGTCCG | *Mus musculus* |
| **mGAPDH** | GTTGTCTCCTGCGACTTCA | GGTGGTCCAGGGTTTCTTA | *Mus musculus* |
| **m18SrRNA** | AGAAACGGCTACCACATCCAA | GGGTCGGGAGTGGGTAATTT | *Mus musculus* |
| **mTLR2** | CGCCCTTTAAGCTGTGTCTC | CGTCAAAGAGCCTGAAGTGG | *Mus musculus* |
| **mTLR4** | CCAACATCATCCAGGAAGGC | GGACTTCTCAACCTTCTCAAG | *Mus musculus* |
| **mCXCL2** | GCCTGAAGACCCTGCCAAG | AACCAGGGGGGCTTCAGGG | *Mus musculus* |
| **mTNFa** | ACGCTCTTCTGTCTACTGAAC | TTGTCTTTGAGATCCATGCC | *Mus musculus* |
| **mIL1b** | GATCCCAAGCAATACCCAAAG | CTTTGTGCTCTGCTTGTGAGG | *Mus musculus* |
| **mNOS2** | GCAGCACTTGGATCAGGAAC | ACCATCTCCTGCATTTCTTCC | *Mus musculus* |
| **mIL6** | AGCCAGAGTCCTTCAGAGAG | GTCCTTAGCCACTCCTTCTG | *Mus musculus* |
| **mCXCL9** | CTGTTCTTTTCCTCTTGGGCA | GGCAGGTTTGATCTCCGTTC | *Mus musculus* |
