# Supplementary material for: Compliant Substrates Enhance Macrophage Cytokine Release and NLRP3 Inflammasome Formation During Their Pro-Inflammatory Response
Source: Front Cell Dev Biol. 2021 Mar 29;9:639815. doi: 10.3389/fcell.2021.639815 (PMC8039395; doi:10.3389/fcell.2021.639815)

Supplementary Material

**Supplementary Figure 1. Relative gene expression levels of pro-inflammatory genes in non-treated macrophages and in cells primed with 100 ng/ml of LPS for 6 h.**

Three independent experiments were conducted with cells from three different mice. Triplicates were done in each experiment. Relative expression was calculated as 2^(-ΔΔCT)^ (relative to the geometric mean of housekeeping genes β-*actin*, *Gapdh* and *18S* rRNA and plastic controls. Mean ± SEM are shown and each dot represents an independent experiment. Statistical analysis was performed using a t-test. **p < 0.01.


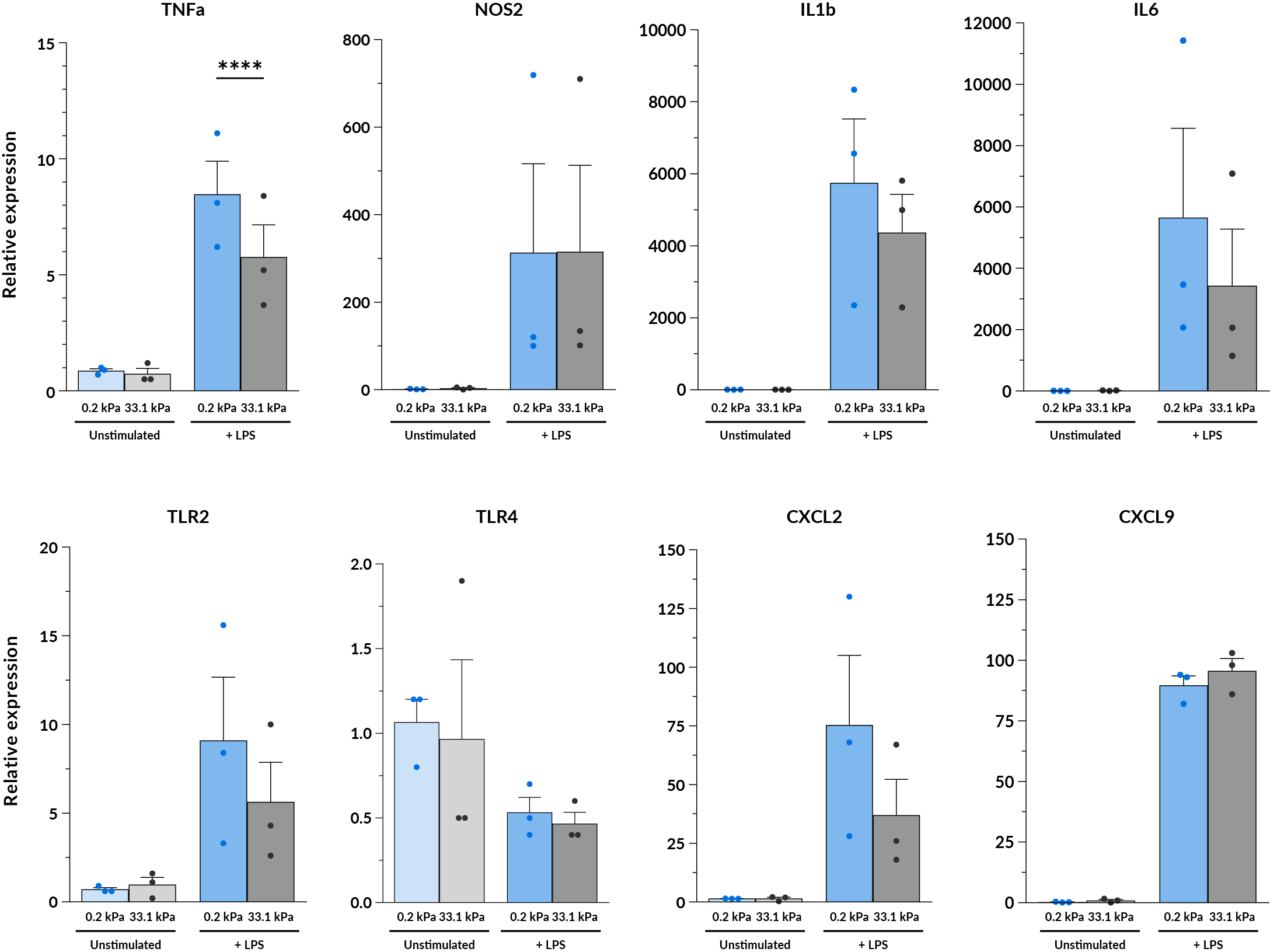


**Supplementary Figure 2. Asessment of cell viability at 0, 60, 90 and 360 min after macrophage priming and stimulation.**

As in Fig. 3E, cell viability was determined by LDH assay for control macrophages cultured on gels and for cells treated with LPS and nigericin. The time axis indicates the minutes after nigericin addition. Mean ± SEM are shown and data corresponds to 2 independent experiment with 3 replicates per condition.


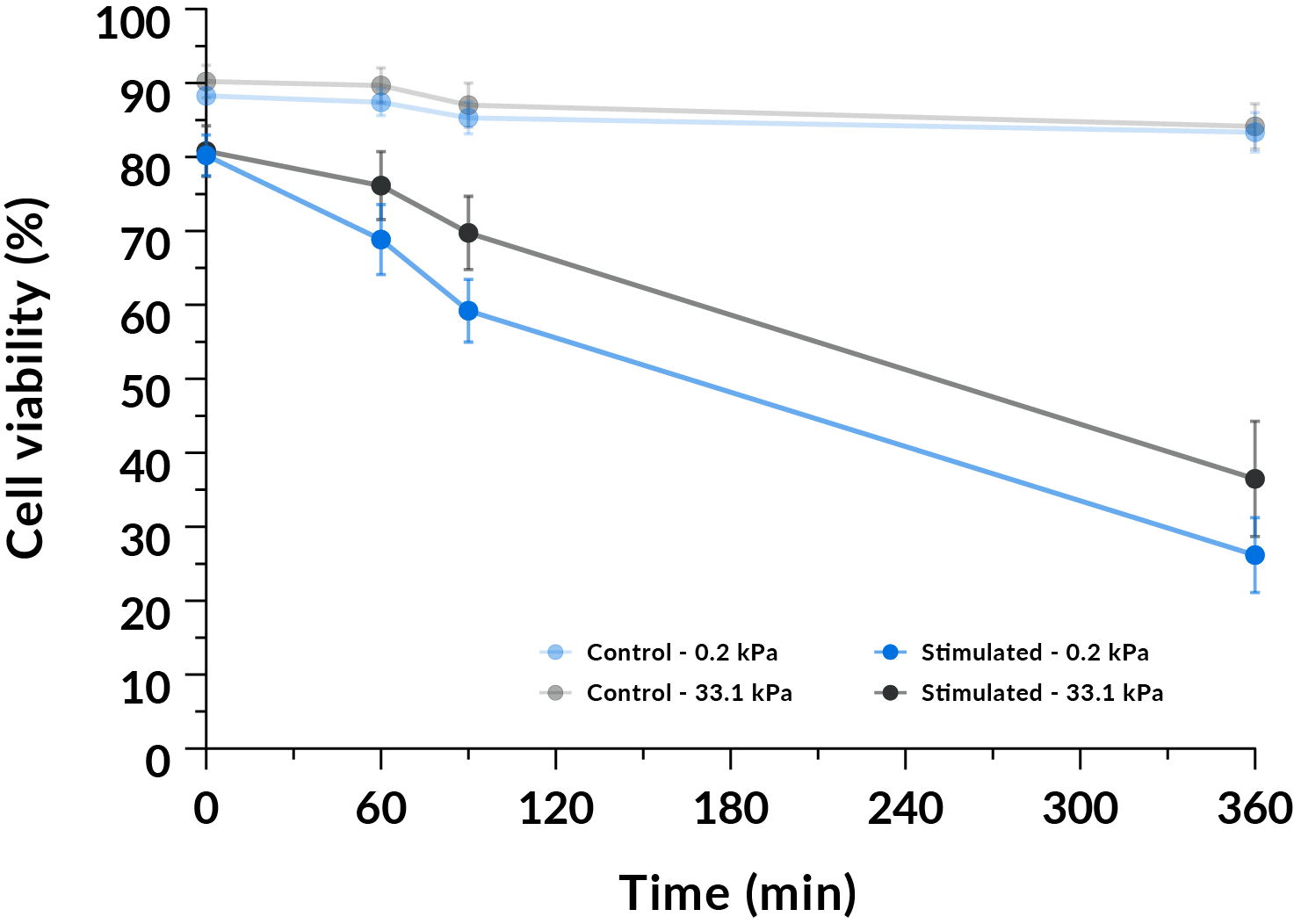


**Supplementary Figure 3. Cell area quantification of ASC speck positive (+) and negative (-) BMDMs on compliant and stiff hydrogels.**

Cells were primed with 100 ng/ml LPS for 4.5 h and stimulated with 10 µM nigericin for 1.5 h and then nuclei, F-actin and ASC were immunostained as in Fig. 3E. Box-plots represent the median and percentiles 25 and 75, and whiskers are the percentiles 10 and 90. Statistical analysis was performed using a Kruskal-Wallis ANOVA followed by Dunn’s post hoc analysis to obtain the multiple comparison p-values. ****p < 0.0001, ns, not significant.


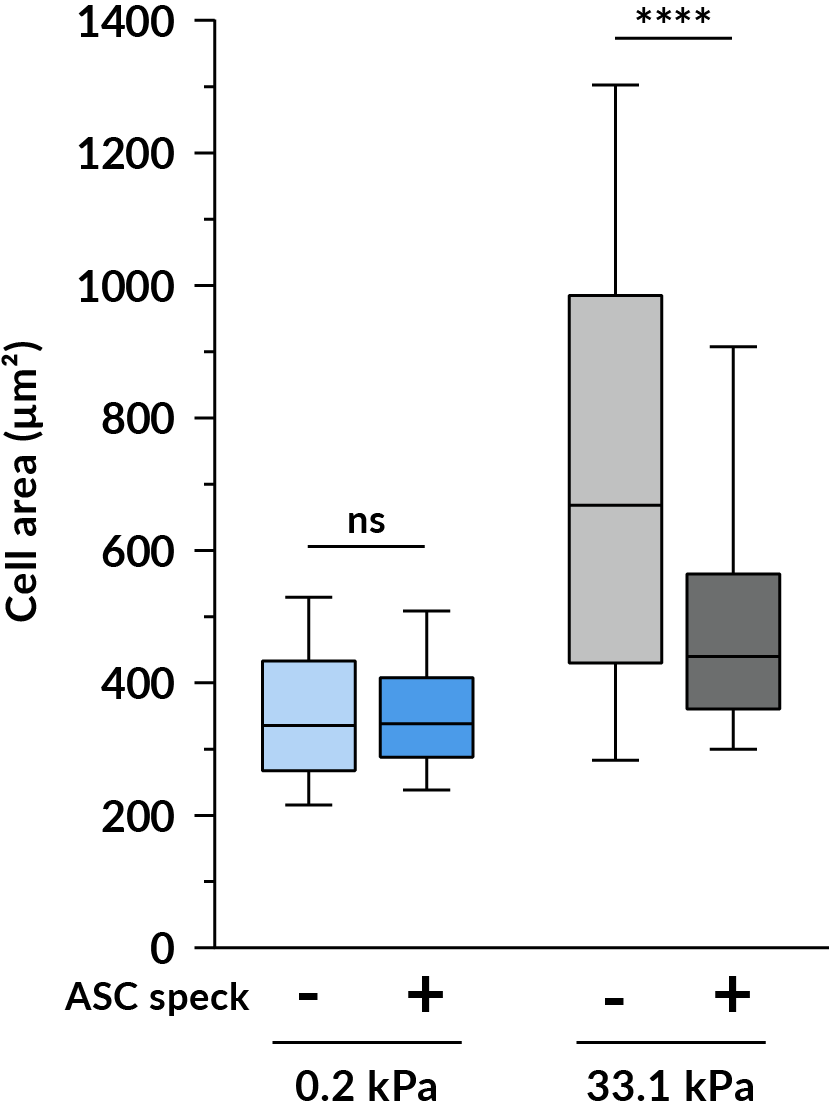


**Supplementary Figure 4. Cell area quantification of ASC speck positive (+) and negative (-) BMDMs on compliant (A) and stiff (B) hydrogels treated with actomyosin inhibitors.**

Cells were pre-treated for 1 h with the either 1:1700 DMSO as control, 10 µM blebbistatin or 10 µM Y-27632. While still keeping the inhibitors, they were then primed with LPS and stimulated with nigericin and then nuclei, F-actin and ASC were immunostained as in Fig. 4C. Box-plots represent the median and percentiles 25 and 75, and whiskers are the percentiles 10 and 90. Statistical analysis was performed using a Kruskal-Wallis ANOVA followed by Dunn’s post hoc analysis to obtain the multiple comparison p-values. Comparisons for each indicated condition were made to the respective DMSO control. ***p < 0.001, ****p < 0.0001, ns, not significant.


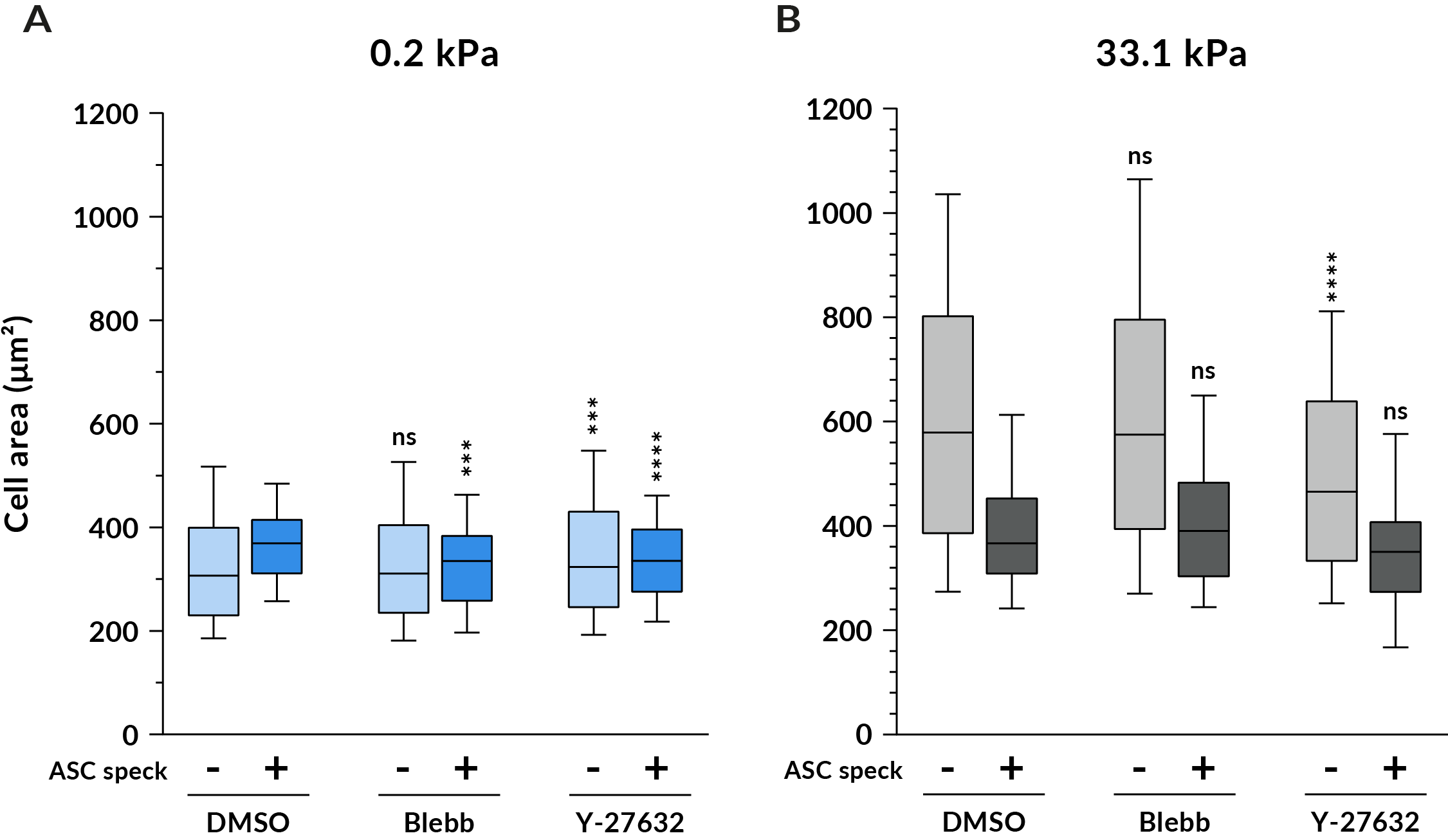

Supplement: Supplementary file 2 [file Table_2.docx]
